# Child DNA methylation in a randomised controlled trial of a video-feedback intervention to promote positive parenting and sensitive discipline (VIPP-SD)

**DOI:** 10.3389/frcha.2023.1175299

**Published:** 2023-04-26

**Authors:** Elizabeth C. Braithwaite, Jessica Cole, Christopher Murgatroyd, Nicky Wright, Christine O’Farrelly, Beth Barker, Paul Ramchandani

**Affiliations:** ^1^ Department of Psychology, Faculty of Health and Education, Manchester Metropolitan University, Manchester, United Kingdom; ^2^ School of Healthcare Science, Faculty of Science and Engineering, Manchester Metropolitan University, Manchester, United Kingdom; ^3^ Centre for Research on Play in Education, Development, and Learning, Faculty of Education, University of Cambridge, Cambridge, United Kingdom

**Keywords:** behavioural problems, developmental psychopatholgy, parenting, DNA methyaltion, intervention

## Abstract

**Introduction:**

A major modifiable risk factor for behavioural difficulties is harsh and insensitive parenting, and it has been hypothesised that the biological mechanism by which parenting influences child behaviour is *via* changes in the child's DNA methylation. We attempted to, in part, address the hypothesis that parenting is associated with child DNA methylation and, in turn, behaviour.

**Methods:**

Primary caregivers of young children with behavioural difficulties (children aged 12–36 months) were randomised to receive a video-feedback Intervention to promote Positive Parenting and Sensitive Discipline (VIPP-SD) (*n* = 151), or usual care (*n* = 149). Child buccal samples were collected at a 2-year post-randomisation follow up (children aged 3–5 years, VIPP-SD group *n* = 106, usual care group *n* = 117) and were assessed for DNA methylation at the NR3C1, FKBP5 and OXYR genes. Child behaviour was assessed at baseline, post-intervention and 2-years post-randomisation using the Preschool Parental Account of Children's Symptoms (PPACS). We examined group differences in DNA methylation, associations of DNA methylation with behaviour, and sex differences.

**Results:**

For the NR3C1 and OXYR genes, there were no group differences, sex differences, or associations of DNA methylation with child behaviour, though all non-significant findings were in the hypothesised direction. For FKBP5 DNA methylation, there was a significant interaction between group and sex, such that males in the usual care group had higher DNA methylation than females, but in the intervention group females had higher DNA methylation than males. However, FKBP5 DNA methylation was not associated with behaviour in males or females.

**Discussion:**

We provide the first evidence from a randomised controlled trial focused on improving parenting for sex-specific changes in child DNA methylation at a key gene involved in stress reactivity and psychopathology. This study adds to our understanding of causal mechanisms linking parenting with child behaviour, which is important for developing targeted interventions. A key limitation is that child DNA methylation was only assessed at one time point, so we were unable to assess change in DNA methylation over time. However, we demonstrate that is possible to collect and analyse DNA samples from families with young children receiving parenting interventions in the community, providing impetus for further research on this topic.

## 1. Introduction

Enduring behavioural problems in childhood are an important early risk factor for conduct disorders, and other psychopathologies in adulthood such as depression, drug and alcohol misuse and antisocial personality disorder (1), and can present multiple challenges across the lifespan (2). To illustrate, 25%–60% of all adult disorders can be traced back to juvenile disruptive behaviours (3). In childhood, disruptive behaviours are characterised by disobedience, angry or irritable mood, and verbal or physical aggression towards others (Diagnostic and statistical manual (DSM-V); APA (4)). Behaviour problems are a source of immediate distress for children and families as well as having long-term costs for peer relationships (5, 6) and engaging positively in school (7, 8). Most children who exhibit disruptive behaviours experience challenges with regulating their emotions and behaviours; an early marker of impulsivity and low self-regulation capability (9). An inability to self-regulate one's emotions is also associated with heightened risk for later substance abuse, health problems, financial hardship, and delinquency (10, 11). Thus, disruptive behaviours in childhood come at a great cost for both individuals, families and society as a whole; therefore, attempts to tackle disruptive behaviours early could yield huge benefits for families, as well as significant economic gains (12). The current study is nested within a randomised controlled trial (RCT) to promote positive parenting and sensitive discipline, which has been shown to improve behavioural outcomes in young children. The aim of the current study was to examine group differences (intervention vs. control) in child DNA methylation and associations with child behaviour, to attempt to elucidate causal epigenetic mechanisms linking parenting and child behavioural difficulties.

Effective prevention strategies for childhood behavioural difficulties rely on our ability to identify the underlying aetiology, and then apply targeted prevention/intervention strategies. A key modifiable risk for behavioural problems in childhood is the parental care that children receive (13). In particular, harsh and physical disciplinary strategies, and low parental sensitivity and responsiveness, have been associated with an increased risk of externalising behaviours (behavioural difficulties, conduct problems and attention difficulties) (13, 14), and functional physiological changes in the autonomic nervous system (ANS) and hypothalamic-pituitary-adrenal (HPA) axis (15) in children. Alterations in the child's stress response systems may reflect a short-term adaptive response to harsh parenting, but in the long term may lead to increased allostatic load (i.e., the cumulative burden of chronic stress and life events) on the neuroendocrine, immune, metabolic, cardiovascular and respiratory systems (16, 17), resulting in system impairments. For example, children in supportive parenting contexts clearly exhibit a quick stress response followed by a recovery and return to resting state. However, in the context of chronic and severe dysfunctional parenting, children's stress response becomes less flexible in response to acute stress (18, 19) demonstrated by blunted (20, 21), or in some cases exaggerated (22), glucocorticoid responses. In children, dysregulated stress reactivity has also been related to behavioural difficulties and conduct disorders (23, 24). However, a key unanswered question is whether epigenetic mechanisms may mediate associations between dysfunctional parenting and behavioural difficulties/dysregulated stress reactivity in children (25).

Epigenetic modifications are biochemical modifications of the DNA that influence gene expression without altering the DNA sequence itself, and DNA methylation is the most widely studied epigenetic mechanism in humans. Cytosine-phosphate-guanine (CpG) sites within the DNA can become methylated with the addition of a methyl molecule (CH_3_), which can occur within the gene sequence or, more commonly, at the promotor region of the gene (26). DNA methylation in the promotor region of a gene, in most cases, prevents DNA transcription, leading to a downregulation or silencing of gene expression (26). This is therefore a biological mechanism by which family experiences, or indeed any experiences or exposures, may become biologically embedded and influence an organism's phenotype.

It has been hypothesised that dysfunctional parenting may lead to the methylation of genes that code for the functioning of children's glucocorticoid stress reactivity system, leading to diminished stress reactivity; a risk factor for behavioural difficulties (25). Initial evidence for this hypothesis is based on pioneering animal research which has robustly demonstrated that low maternal care is associated with altered DNA methylation of the glucocorticoid receptor (GR) gene (*nr3c1*), as well as GR gene expression and HPA stress responses, in offspring (27, 28). Evidence for comparable mechanisms in humans is clear. It is well established in humans that exposure to early life stress is associated with hypermethylation of the NR3C1 gene which encodes the glucocorticoid receptor (29, 30). NR3C1 hypermethylation has also been associated with emotion regulation difficulties and externalising behaviour (31), and altered HPA reactivity (30, 32) in children. There is also evidence for sex differences in DNA methylation of NR3C1 in relation to early life adversity and child behavioural and emotional outcomes (33), with girls at greater risk of developing externalising symptoms and poor emotional outcomes, mediated by changes in DNA methylation.

In addition to NR3C1, other candidate genes which play a role in glucocorticoid stress responses have also been examined in relation to early life stress and child behavioural and physiological outcomes. There is an accumulating literature which has focused on the *FKBP5* gene, which codes for the FK506-binding protein 51 (FKBP5). FKBP5 is a co-chaperone for the GR receptor which modulates its sensitivity and is involved in the HPA-axis negative-feedback loop (34). There are several polymorphisms within the *FKBP5* gene which appear to moderate effects of early life stress on psychopathology (35), with “T” allele carriers more at risk of depression and PTSD (36) and alterations in DNA methylation (37) following early adversity. Maltreatment has been associated with reduced DNA methylation in the promoter region of the FKBP5 gene in children (38). Conversely in adults, reduced DNA methylation at the FKBP5 gene has been associated with a greater response to psychological therapy for agoraphobia, and reduced anxiety following treatment (39). However, developmental changes in the epigenome are unclear therefore comparing DNA methylation of children to adults is challenging.

Another biological system which has been the target of research concerning mechanisms of how the early environment can impact phenotype is the oxytocin system. The human oxytocin system is essential to the regulation of complex social behaviours and is also implicated in psychopathologies characterised by social deficits (40, 41). Emerging evidence suggests that variation in the epigenetic regulation of the oxytocin receptor gene (*OXYR*) provides the oxytocin system with the flexibility to respond to environmental factors, especially those that occur during childhood (41). Robust evidence from animal studies has demonstrated that poor maternal care in prairie voles is associated with increased DNA methylation at the *oxyr* gene and decreased expression of the oxytocin receptor in the nucleus accumbens (42). Additionally, treatment of mandarin vole pups with an oxytocin antagonist resulted in decreased attachment behaviours of the pups towards the dams (43); providing experimental evidence that reduced oxytocin signalling (which also occurs in the case of OXYR hypermethylation) directly impacts attachment behaviour. In humans, it is evident that oxytocin is important in early parent-infant interactions; elevated parent and child oxytocin is associated with more parent-infant contact, and also elevated parental oxytocin is associated with more responsive parenting (44). Adults, however, who retrospectively reported low levels of maternal care in childhood have elevated OXYR DNA methylation in peripheral blood (45), suggesting long-term impacts of parenting behaviours on the epigenetic regulation of children's oxytocin system. Critically, increased OXYR DNA methylation has been associated with callous unemotional traits and difficulties with affect regulation in children (46).

Accumulating evidence therefore implicates the role of DNA methylation mechanisms at the glucocorticoid stress response and oxytocin systems as a potential mediator of the link between parenting behaviours and child psychopathology. However, longitudinal evidence that directly links parenting with *both* child DNA methylation and behaviour is lacking. Additionally, it is currently unknown whether interventions that target parenting can reverse changes in child DNA methylation, and whether this will lead to long-term reductions in disruptive behaviour. To address this evidence gap, randomised controlled trials where parenting is manipulated are needed.

Here, we present data from a randomised controlled trial of a home-based video-feedback intervention to promote positive parenting and sensitive discipline (VIPP-SD) in parents with the aim of reducing behaviour problems in children aged 12–36 months (*N* = 300). The trial was effective at reducing behaviour problems in the children at the 5-month post-intervention follow up (47). At the two-year follow up, buccal samples (*n* = 225) were collected from the children to examine DNA methylation at the NR3C1, FKBP5 and OXYR genes. The aims of the current study were to examine (A) whether there were group differences (intervention vs. usual care) in DNA methylation, and (B) if child behavioural difficulties were associated with DNA methylation. Because of the evidence of sex differences in effects of early life stress on psychopathology and DNA methylation at the NR3C1 gene, an additional aim was to examine sex differences in all analyses, but these were exploratory, and we did not make specific *a priori* hypotheses concerning sex differences. Our hypotheses were as follows:

H_1_: The intervention group will have reduced DNA methylation at the NR3C1 and OXYR genes, and elevated DNA methylation at the FKBP5 gene, compared with the usual care group.
H_2_: Behavioural problems will be associated with elevated DNA methylation at the NR3C1 and OXYR genes, and reduced DNA methylation at the FKBP5 gene.

## 2. Methods

### 2.1. Participants and procedures

Data derives from a two-arm, parallel group, researcher-blind, randomised-controlled trial (RCT) to test the clinical and cost-effectiveness of a brief video-feedback psychological intervention aimed at improving positive parenting and sensitive discipline [VIPP-SD (48, 49),] for parents of young children (aged 12–36 months) at risk of behavioural difficulties (ISRCTN58327365) (47, 50, 51). VIPP-SD is a manualised, home-based intervention, delivered over six sessions of 1- to 2-hour duration at approximately fortnightly intervals. The intervention was provided in the community and delivered by trained health practitioners. Each session had two parts: the first part involved filming parent-child interactions, and the second part involved giving parents focused feedback based on the filmed interactions from the previous sessions. For more information on the intervention please see O'Farrelly et al. (47).

Participants were 300 families who were randomised to receive either the VIPP-SD intervention (*n* = 151) or treatment as usual (*n* = 149), for details on the sample size calculation and randomisation process please see O'Farrelly et al. (47). Families included young children (aged 12–36 months) who demonstrated emerging behavioural difficulties, and their parents, see O'Farrelly et al. (47) for more information on the recruitment process. Eligibility for inclusion in the trial was as follows: parents aged 18 or over; child aged between 12 and 36 months; child scored in top 20% for behavioural difficulties on the Strengths and Difficulties Questionnaire (SDQ) (52, 53). Families were excluded if: the child or parent had a severe sensory impairment, learning disability, or language limitation that precluded participation in the trial; there were siblings participating in the trial; families were participating in active family court proceedings; parent/carer was participating in another closely related research trial and/or was currently receiving an individual video-feedback-based intervention. Participants in both groups continued to receive their usual care, which was minimal in most cases (there are no standard care pathways in the NHS for early-onset behaviour problems). Some participants received support and advice from a health visitor or GP, referral to early intervention mental health services linked to a children's centre, or parenting advice and support sessions. Data were collected on the concurrent use of health and social care services.

Assessments were conducted at baseline, and at 5- and 24-months post-randomisation and were completed by researchers who were blind to the family's treatment status (51). Baseline and 5-month follow-up data were collected between July 2015 and April 2017, and 24-month follow-up data was collected between October 2017 and July 2019. The primary outcome was an assessment of severity of behavioural problems using a modified version of the Preschool Parental Account of Children's Symptoms (PPACS), a semi-structured investigator-led interview administered to the child's primary caregiver (54, 55). Child behaviour was also assessed at each time point using the SDQ and the Child Behaviour Checklist (CBCL) (56), as well as the PPACS. Demographic information was collected at baseline. More details of the measures are available in the trial protocol (50) and publication (47).

Buccal samples were collected from the children participating in the trial at the 24-month post-randomisation follow up using the iSwab-DNA-250 collection device (Mawi, UK) (*N* = 225, 75% of the trial sample). We used this method to collect child DNA samples because it allows the storage of DNA samples at room temperature. Attrition in sample size from the full trial sample was because of study drop-out (*n* = 14), because caregivers did not provide consent to the collection of DNA samples from their children (*n* = 17), because children did not provide assent for the collection of the sample (*n* = 26), and because some follow-up assessments were conducted over telephone only so no in-person contact with participants occurred to collect the sample (*n* = 18). Families who provided a child DNA sample were not statistically different to the whole sample on any of the demographic measures used as confounders in the analyses (all *p*'s < 0.05). Samples were stored at room temperature at Imperial College London for 20 months and were then transported to Manchester Metropolitan University for analysis, which was conducted between May and November 2021.

The trial protocol was approved by Riverside Research Ethics Committee (14/LO/2071) as part of the NHS Research Ethics Service for more details see O'Farrelly et al. (47). Parents or caregivers provided informed consent, and the trial followed the Consolidated Standards of Reporting Trials (CONSORT) reporting guidelines. Additional ethical approval was gained from the Manchester Metropolitan University Research Ethics Committee (REF 10452) prior to the transportation of buccal samples from Imperial College London to Manchester Metropolitan University.

### 2.2. Measures

#### 2.2.1. Demographics

Primary caregivers reported demographic characteristics at baseline. These included: child sex (male/female), date of birth, and race/ethnicity (asian/black/mixed/other/white), and the primary caregivers' sex (male/female), age (in years), race/ethnicity (asian/black/mixed/other/white), employment status (employed/paid parental leave/self-employed/student/looking after home and children) and highest educational qualification (GCSE or lower/A level, NVQ, or BTEC/University graduate or postgraduate degree). For more details please see (47).

#### 2.2.2. Child behaviour

Three parental reports of child behaviour were collected as part of the trial at baseline and at the 5-month and 24-month follow up assessments: the PPACS, the SDQ and the CBCL. In the current analysis we chose to only use data from the PPACS to minimise the number of statistical tests and to reduce the likelihood of reporting a false-positive result. We chose the PPACS because it was the primary outcome used in the trial. The PPACS is a semi-structured researcher-led interview administered to a parent or caregiver (55). Interviews are the criterion standard outcome measure as they provide a more complete picture of children's symptoms that it is possible to measure by questionnaire (57, 58). To determine scores, the primary caregiver provided detailed examples of the child's typical behaviour over the last week in a range of settings (e.g., in the home, with friends, in public). The objective of this approach is to allow the interviewer to rate the child's behaviour based on real examples, rather than the caregiver's global impressions or judgements of whether or not the behaviour is normal. To ensure that the example given is characteristic of the child, caregivers are asked how representative the described behaviour is of the child over the past 4 months. A trained interviewer then rated the severity and frequency of the symptoms based on their professional judgement, following training, and guided by written definitions and thresholds of each of the scored behaviours. The measure comprises two subscales: conduct problems and attention-deficit/hyperactivity disorder or hyperactivity. In this study we used the total score in all analyses. The PPACS has high inter-rater reliability and good construct validity, and has been used in several RCTs assessing intervention effects on child behaviour (55, 59, 60). Interviews were recorded, and 10% (30 out of 300) were randomly selected for double scoring at each time point; high reliability was observed (intraclass correlations 0.93–0.97).

#### 2.2.3. Child NR3C1 1F, FKBP5 and OXYR DNA methylation

##### 2.2.3.1. DNA isolation

DNA from the buccal samples was extracted using DNA extraction kits (Quiagen, UK) in accordance with the manufacturer's protocol, and the salivary DNA was quantified using a Nanodrop 1,000 spectrophotometer (Thermo Scientific, UK). The extracted DNA was in the range 9.3–114.8 ng/µl (mean = 46.12, SD = 20.69). Extracted DNA samples were stored at −20°C.

##### 2.2.3.2. Bisulphite pyrosequencing

DNA methylation at specific CpG sites (see Supplementary Figure S1) on the NR3C1, FKBP5 and OXYR genes was analysed using the quantitative bisulfite-pyrosequencing method. The extracted DNA (500 ng) was bisulfite converted using the EpiTect Bisulphite kit (Qiagen Ltd, UK) according to the manufacturer's instructions and stored at −20°C until PCR processing. PCR was performed to amplify the DNA and label it with biotin for pyrosequencing. A mastermix was prepared for each reaction, including: 4 µl of 5x MyTaq reaction buffer (Bioline, UK), 0.5 µl of forward primer and 0.5 µl of reverse primer (see Table 1 for primer sequences), 0.2 µl MyTaq hot start DNA polymerase (Bioline, UK), and 12.8 µl water to make a total solution volume of 18 µl. This solution was vortexed and 18 µl aliquots were added to each well of a 96 well PCR plate. DNA (2 µl) was added to each well, then placed into an Eppendorf thermocycler (94 °C, 1 min; 60 °C, 1 min; 72°C, 1 min; 50 cycles). Electrophoresis of the PCR products (5 µl) was performed to confirm success of the PCR reaction. Pyrosequencing was performed using a PyroMark Q24 pyrosequencer (Qiagen Ltd, UK) with specific pyrosequencing primers using 20 µl of bisulfite-converted DNA. The average DNA methylation levels of specific CpG sites was quantified using PyroMark Q24 2.0.4 software (Qiagen Ltd, UK).

**Table 1:** Forward and reverse primers used for NR3C1, FKBP5 and OXYR, location and sequence size.

Gene	Primers, forward (f), reverse (r)	Location	PCR size (bp)
NR3C1	F-(Biotin)AATTTTTTAGGAAAAAGGGTGG	hg19; chr5:142,783,610–142,783,671	343
	R-AACCCCTTTCCAAATAACACACTT		
FKBP5	F-GGATTTGTTGGGATAATAATTTTGGG	Chr6: 35,558,486–35,558,567	324
	R-(Biotin)TCTTACCTCCAACACTACTACTAAA		
OXYR	F- GGGGGGAGTTAATTTTAGGTT	hg19:Chr:3:8,810,807–8,810,808	330
	R-(Biotin)CTCAATCCCCAAAAATCTTTACAATCT		

##### 2.2.3.3. CpG sites

DNA methylation of two CpG cites in the FKBP5 promoter region previously linked to child maltreatment and response to psychological intervention (37, 39, 61, 62) were assessed. Average methylation at FKBP5 CpG1 was 97.47% and 78.68% at CpG2. Mean methylation of the two CpG sites was used in analysis; previous research has shown similar reductions in DNA methylation at both CpG sites in response to maltreatment (38, 62). Supplementary Figure S1 shows the position of the assessed CpG sites in the FKBP5 gene upstream of the coding region.

Four CpG sites on the NR3C1 promoter region previously linked to child adversity and behaviour (29, 63, 64) were assessed for methylation, see locations in Supplementary Figure S1. For analysis, the mean of CpG1 (average = 8.89%) and 2 (average = 4.50%) methylation was used, an approach that has previously been taken (33, 65). Not all bisulphite samples produced clear enough bands to give high enough peaks in the sequencing for the Pyromark software to consider them accurate enough. Therefore, a smaller sample (compared with other genes) with clean PCR bands of *N* = 139 samples were available for the NR3C1 analyses.

DNA methylation was assessed at two CpG sites within the OXYR gene which have previously been linked to child conduct disorders (66, 67) and maternal care (45, 68), see position in Supplementary Figure S1. The mean DNA methylation at CpG1 (47.57%) and CpG2 (69.05%) were used in separate analyses.

### 2.3. Statistical analysis

Imputation of missing child behavioural data is described in O'Farrelly et al. (47). Analysis was conducted in Stata version 17. Any values on the methylation scores >3 standard deviations (SD) above or below the mean were winsorised to 3 SD above/below the mean. No imputation was conducted for the child DNA methylation data. FKBP5 and NRC31 DNA methylation values were skewed and therefore transformed using log and square root transformation, respectively. The two OXYR DNA methylation variables were approximately normally distributed. All analyses controlled for study site (using Islington as reference), months between randomisation and outcome, age of child at recruitment, number of caregivers participating and baseline PPACS total score. Hypothesis 1 was tested using a 2 (treatment group; intervention/usual care) x 2 (child sex; male/female) analysis of variance with covariates (ANCOVA) models with each of the 4 methylation scores (NR3C1, FKBP5, OXYR CpG1, and OXYR CpG2) as outcomes. Significant interactions were explored and plotted using the margins command to estimate the predicted marginal effects for each combination of predictors (intervention vs. usual care and females vs. males). Main effects of group were then explored using a one-way ANCOVA in males and females separately. Hypothesis 2 was tested using multiple linear regression predicting time 3 PPACS scores from DNA methylation (4 separate models) and infant sex and their interaction term, in the intervention and control groups separately. Bonferroni correction was applied to account for multiple testing, with a threshold of *p* < .006 (0.05/8 statistical tests) set for significance.

## 3. Results

### 3.1. Demographics

Demographic statistics of the sample with DNA methylation data are presented in Table 2, split by treatment group. There were no differences between the intervention and usual care groups on any of the demographic measures (all *p*'s < 0.05). Means and standard deviations of the DNA methylation variables are shown in Table 3.

**Table 2:** Demographic characteristics of the sample, split by intervention and control group.

Characteristic	VIPP-SD Group	Usual Care Group
	*n* = 106	*n* = 117
Children		
Male, *N* (%)	55 (51.9)	67 (57.3)
Age at baseline, mean (SD) in months	22.92 (6.88)	23.44 (6.51)
Age at 2 year follow up mean (SD) in months	47.58 (7.29)	47.92 (6.91)
Race/ethnicity, *N* (%)		
Asian	8 (7.5)	5 (4.3)
Black	3 (2.8)	10 (8.5)
Mixed	28 (26.4)	20 (17.1)
Other	2 (1.9)	6 (5.1)
White	65 (61.3)	76 (65.0)
Primary caregivers		
Male, *N* (%)	2 (1.9)	5 (4.3)
Age, mean (SD) in years	34.39 (4.98)	34.56 (6.07)
Race/ethnicity, *N* (%)		
Asian	13 (12.3)	12 (10.3)
Black	3 (2.8)	10 (8.5)
Mixed	7 (6.6)	8 (6.8)
Other	7 (6.6)	3 (2.6)
White	76 (71.7)	84 (71.8)
Employment status, *N* (%)		
Employed	47 (44.3)	52 (44.4)
Paid parental leave	5 (4.7)	7 (6.0)
Self-employed	14 (13.2)	9 (7.7)
Student	2 (1.9)	5 (4.3)
Looking after children at home	38 (35.8)	44 (37.6)
Highest qualification, *N* (%)		
GSCE or lower	9 (8.5)	10 (8.6)
A level, NVQ, or BTEC	29 (27.4)	28 (23.9)
University graduate or postgraduate degree	68 (64.2)	79 (67.6)

**Table 3:** DNA methylation (% methylation) variables split by intervention and control group.

	VIPP-SD group		Control group	
	*N*	Mean (SD)	*N*	Mean (SD)
NR3C1 (mean CpG1 and CpG2)	66	6.15 (6.78)	73	7.16 (9.05)
FKBP5 (mean CpG1 and CpG2)	104	88.30 (6.56)	115	87.84 (7.75)
OXYR CpG1	106	47.32 (5.14)	116	47.80 (5.73)
OXYR CpG2	106	69.41 (5.33)	116	68.73 (5.39)

### 3.2. Addressing hypothesis 1

**Figure 1:**
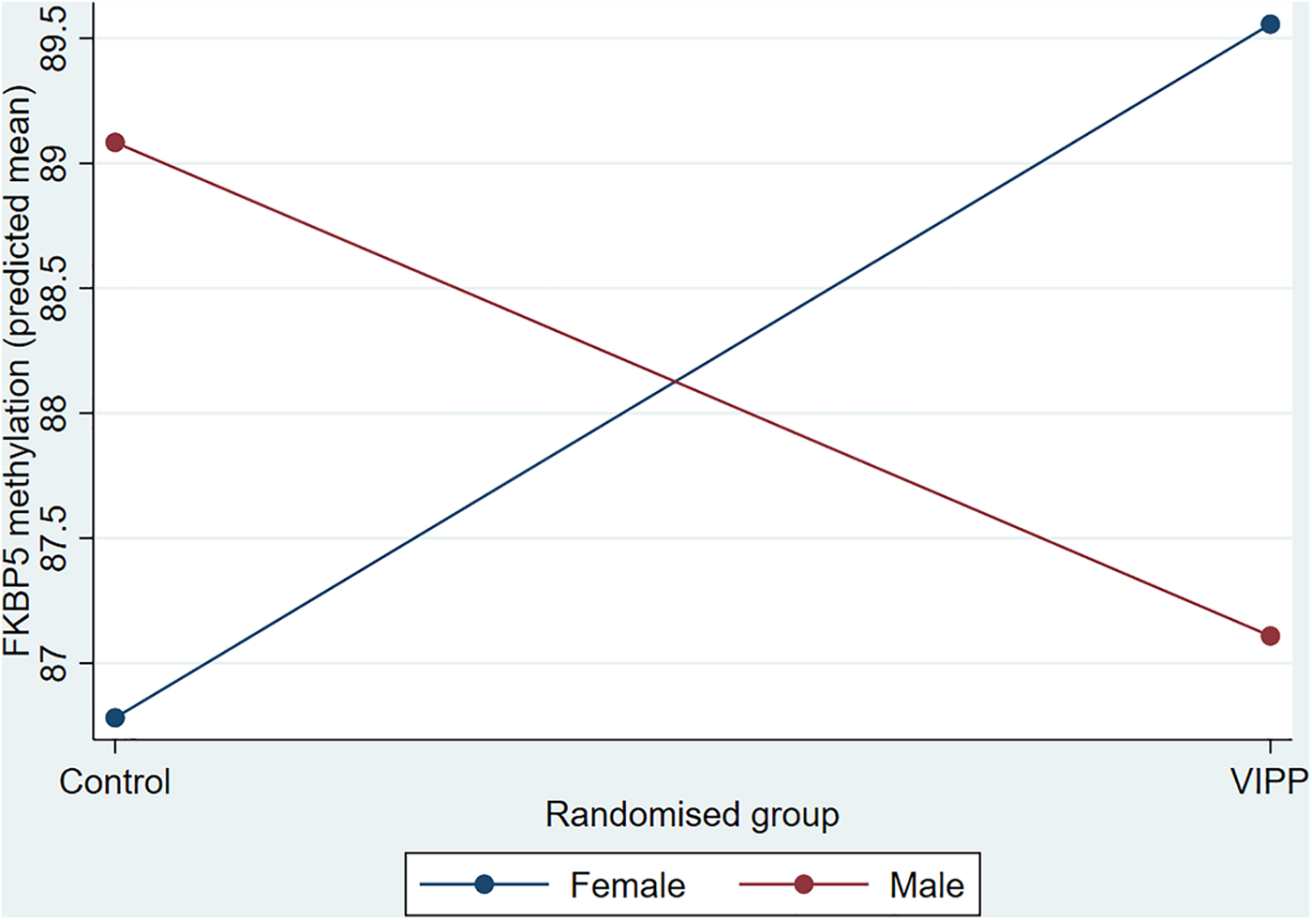
FKBP5 DNA methylation predicted mean by intervention group and child sex.

Hypothesis 1, that treatment group would be associated with methylation, and the exploratory examination of sex differences, was tested using a 2 (treatment group) by 2 (child sex) ANCOVA for each of the 4 methylation scores, accounting for confounders and baseline PPACS symptoms. For OXYR CpG1, OXYR CpG2 and NR3C1 the main effects of group, sex and the interaction term were all non-significant (all *p*'s > 0.006), indicating no association between treatment group, either as a main effect or modified by sex, and DNA methylation at these CpG sites, see Supplementary Table S1. For FKBP5, the main effects of group and child sex were non-significant, but the interaction term was significant [F(13, 202) = 8.42, *p* = 0.004] (see Table 4). The interaction is displayed in Figure 1, which shows the predicted marginal effects for girls and boys in the intervention and usual care groups. The FKBP5 DNA methylation is higher in females in the intervention group (mean = 86.85, SD = 5.91; predicted marginal mean = 82.53, SE = 6.27) than the usual care group (mean = 86.84, SD = 6.81; predicted marginal mean = 79.72, SE = 6.17). Conversely for males, FKBP5 DNA methylation is lower in the intervention group (mean = 87.31, SD = 6.57; predicted marginal mean = 80.16, SE = 6.25) compared to the usual care group (mean = 88.92, SD = 7.13; predicted marginal mean = 82.01, SE = 6.29). The main effect of group was examined in a one-way ANOVA split by child sex. There was a small to medium effect size (partial eta squared = 0.05) for the effect of group in females which was non-significant [F(11,85) = 4.38, *p* = 0.039], and a small effect size in males (partial eta squared = 0.02) which was non-significant [F(11,118) = 1.99, *p* = 0.161].

**Table 4:** Result of the ANCOVA examining the impact of intervention group, child sex, and their interaction on FKBP5 DNA methylation.

FKBP5 DNA methylation		
Variable	F	p
Intervention group	0.00	0.947
Child sex	0.00	0.967
Intervention group X child sex	**8** **.** **42**	**0** **.** **004**
PPACS T1	1.77	0.184
Child age	1.98	0.161
Number of caregivers in trial	0.66	0.419
Time since randomisation	7.10	0.008
Location—Camden	1.16	0.282
Location—Hillingdon	5.45	0.021
Location—Oxford	3.28	0.072
Location—Barking	3.13	0.079
Location—Peterborough	3.59	0.059
Location—Hertfordshire	0.05	0.831
Model *N* = 217, R-squared = 0.119		

### 3.3. Addressing hypothesis 2

The second hypothesis, that methylation would be associated with behavioural difficulties at the 24-month follow up, with the possibility of sex differences, was examined using multiple linear regression predicting PPACS total score at the 24-month follow up from treatment group, child sex, and the interaction term between them, after accounting for confounders and baseline PPACS symptoms. OXTR CpG1, OXTR CpG2, NR3C1 and FKBP5 DNA methylation were not significantly associated with PPACS total behavioural difficulties in the intervention or usual care group, see Supplementary Table S2.

## 4. Discussion

This study aimed to further scientific understanding of whether parenting can impact children's DNA methylation, which in turn impacts behavioural difficulties. Our first aim was to test whether there were group (intervention group vs. usual care group) and/or sex (male vs. female) differences in child DNA methylation at the NR3C1, FKBP5 and OXYR genes. There were no main effects of group, sex, or their interaction on DNA methylation at the NR3C1 and OXYR genes. However, there was a significant interaction between group and sex on FKBP5 DNA methylation, after applying a stringent statistical control for multiple testing. Further analysis established that females in the intervention group had higher DNA methylation than females in the control group, whereas males in the intervention group had lower DNA methylation than males in the control group. There were no associations between child DNA methylation at any genes and child behaviour.

We hypothesised that the intervention group would have higher FKBP5 DNA methylation than the control group. This was based on evidence that child maltreatment has been associated with reduced DNA methylation at this gene (38), therefore we followed the hypothesis that if there were improvements in parenting (and using the intervention group compared to the usual care group as a proxy for this), then the hypomethylation of this gene in children may be reversed. Results are therefore in the hypothesised direction for females, but in the opposite direction for males. Our examinations of sex differences were exploratory, not determined *a-priori*, and based on evidence from studies implicating sex differences in effects of early life stress on DNA methylation at the NR3C1 gene (33, 69). Therefore, the sex difference in effect of treatment group on FKBP5 DNA methylation was not directly hypothesised and should be interpreted with caution. That said, both the glucocorticoid receptor and FKBP5 play a key role in moderating stress responses and mediating risk for psychopathology. As far as the authors are aware, this is the first evidence for the sex-specific impact of parenting on FKBP5 DNA methylation, and it requires replication. There is however evidence from studies of adults of female-specific associations between FKBP5 DNA methylation and bedtime cortisol (70), and between FKBP5 mRNA expression and symptoms of depression and anxiety (70). Our findings, alongside existing research, therefore support the idea that FKBP5 DNA methylation may be of particular importance in stress reactivity and psychopathology in females, and our findings also implicate DNA methylation at this gene as being malleable to changes in parenting. However, it is evident that polymorphisms within the FKBP5 gene moderate effects of environmental stress on psychopathology (35, 36) and DNA methylation (37), therefore future studies should consider the interaction between genotype and DNA methylation in mediating risk. In the current study we were unfortunately unable to determine and control for FKBP5 genotype.

Our second aim was to examine whether DNA methylation at the NR3C1, FKBP5 and OXYR genes was associated with behavioural difficulties 2 years post-intervention, whilst controlling for baseline (pre-intervention) behaviour. We found no evidence to suggest that DNA methylation was associated with change in behavioural difficulties from baseline to 2 years post-intervention. This is in contrast to previous literature which has examined associations between NR3C1 DNA methylation and externalising behaviour (31), and OXYR DNA methylation and callous unemotional traits and affect dysregulation in children (46, 66). Discrepancies could be explained by comparisons with larger cohorts which included severely maltreated children (31), or because of comparisons with a samples of children with severe behavioural disorders (e.g., callous unemotional traits) (66). The sample of children in the current study were demographically low-risk, and had moderate behavioural difficulties at a very young age, which could explain why there were no associations between DNA methylation and behaviour, as reported in other studies.

Understanding the impact of the early environment on child DNA methylation and psychopathology is an emerging field. There is preliminary evidence that parenting is associated with child DNA methylation (25), and that DNA methylation is associated with child and adolescent psychopathology (71). However, most of the existing work is correlational in nature and few studies have examined DNA methylation in relation to *both* parenting and child behaviour/psychopathology. One very small study of just 23 maltreated children showed that an intervention to enhance caregiving, Attachment and Biobehavioural Catchup (ABC), resulted in genome-wide variation in DNA methylation in those children who received the intervention (*n* = 12) compared with those who did not (*N* = 11) (72). Although promising, these results should be interpreted with caution given the small sample size and the whole-genome approach where issues of multiple-testing are difficult to address. The current study, therefore, extends existing knowledge by examining DNA methylation in children in the context of a fully-powered, randomised controlled trial (RCT) aimed at improving positive parenting and sensitive discipline, and testing associations with child behavioural difficulties. Research of this kind, nested within an RCT, provides the strongest evidence of causal relationships between parenting, child DNA methylation and behaviour. A major strength of this study is the collection of child DNA within an RCT design, which extends existing methodology by demonstrating that it is possible to collect buccal samples from young children to be analysed for DNA methylation within an RCT delivered in the community. If future studies adopt this approach, then more evidence on causal pathways will accumulate to further advance our knowledge. Another strength of this study is that the measure of child behavioural difficulties is based on a parental interview of child behaviour, which allowed the collection of detailed information about symptoms based both on severity and frequency that was not weighted by the parent but by the research team based on strict criteria (55). This measure of child behaviour therefore minimises reporter bias, which is often a limitation of observational research focused on child behaviour.

There were also limitations to the current study that should be considered. First, due to a technical issue with equipment over a period of a COVID-19 lockdown in the UK, the data for the DNA methylation at the NR3C1 gene is incomplete and therefore there is a reduced sample size for analyses of this gene. Thus, power to detect small to medium effects was reduced in this analysis. Overall, the study had a moderate sample size and was underpowered to detect small effects. Second, we did not have data on DNA methylation at the candidate genes prior to the intervention so were unable to assess change in DNA methylation over time. We were therefore unable to directly test the hypothesis that there would be greater changes in child DNA methylation in the intervention compared to the control group, and future work should seek to establish this. Third, assessment of DNA methylation from buccal swabs is limited because it does not necessarily reflect DNA methylation in brain tissue. Fourth, we were unable to control for FKBP5 genotype, as discussed previously. Fifth, we were also unable to directly test the proposed causal mechanism that changes in parenting results in changes in DNA methylation, and ultimately a change in child behaviour. Instead, we used trial group as a proxy for parenting behaviour, with the assumption that those caregivers in the intervention group would show a change in positive parenting and sensitive discipline over time, whereas the caregivers in the control group would show no change in parenting. Future work should seek to assess changes in parenting using observational methods, such as sensitivity and responsiveness, pre- and post-intervention to directly test the proposed causal mechanism. Additionally, replication of these findings using larger, more diverse samples is needed, with rigorous control for confounding variables and potential gene-environment interactions.

In sum, we provide novel evidence, from a fully powered RCT aimed at improving positive parenting and sensitive discipline, that there are impacts of the intervention on child DNA methylation at the FKBP5 gene, consistent with sex-specific effects. We also demonstrate that it is possible to collect and analyse child DNA samples within an RCT delivered in the community to assess levels of DNA methylation; an objective outcome from a parenting intervention that is not subject to the limitations of self-report or observational measures. Research of this type is needed to fully understand causal pathways linking parenting with child DNA methylation and behaviour. Whilst considering the limitations of this study, this work provides impetus for more research on this topic to fully understand how parenting practices may become biologically embedded, resulting in long term consequences for child behaviour and psychopathology.

## Data Availability

The datasets presented in this article are not readily available. All of the individual, de-identified participant data will be available 12 months after publication and for 5 years after date of publication. Data will be made available to researchers who provide a methodologically sound proposal and have the required institutional approvals in place to achieve aims in the approved proposal. Proposals should be directed to the corresponding author to gain access, and requestors will be asked to sign a data access agreement. Requests to access the datasets should be directed to EB, e.braithwaite@mmu.ac.uk.
