# Supplementary material for: Child DNA methylation in a randomised controlled trial of a video-feedback intervention to promote positive parenting and sensitive discipline (VIPP-SD)
Source: Front Child Adolesc Psychiatry. 2023 Apr 26;2:1175299. doi: 10.3389/frcha.2023.1175299 (PMC11731625; doi:10.3389/frcha.2023.1175299)
Supplement: Supplementary file 1 [file Table1.docx]

Supplementary Table 1, Results of the ANCOVA analyses examining NR3C1 and OXYR DNA methylation by intervention group, sex and their interaction.

| **NR3C1 DNA methylation** | | | **OXYR CpG1 DNA methylation** | | | **OXYR CpG2 DNA methylation** | | |
| --- | --- | --- | --- | --- | --- | --- | --- | --- |
| **Variable** | F | p | **Variable** | F | p | **Variable** | F | p |
| Intervention group | 1.17 | 0.281 | Intervention group | 0.28 | 0.6 | Intervention group | 1.05 | 0.307 |
| Child sex | 0.93 | 0.336 | Child sex | 0.42 | 0.519 | Child sex | 0.14 | 0.705 |
| Intervention group * child sex | 0.11 | 0.744 | Intervention group * child sex | 0.39 | 0.533 | Intervention group * child sex | 0.53 | 0.468 |
| PPACS T1 | 0.00 | 0.944 | PPACS T1 | 0.20 | 0.654 | PPACS T1 | 0.12 | 0.724 |
| Child age | 1.75 | 0.189 | Child age | 0.05 | 0.826 | Child age | 1.44 | 0.231 |
| Number of caregivers in trial | 0.05 | 0.825 | Number of caregivers in trial | 0.48 | 0.491 | Number of caregivers in trial | 0.41 | 0.523 |
| Time since randomisation | **13.07** | **<0.001** | Time since randomisation | 6.21 | 0.014 | Time since randomisation | 3.52 | 0.062 |
| Location - Camden | 0.09 | 0.763 | Location - Camden | 0.02 | 0.886 | Location - Camden | 0.03 | 0.859 |
| Location - Hillingdon | 0.42 | 0.517 | Location - Hillingdon | 0.03 | 0.866 | Location - Hillingdon | 2.23 | 0.635 |
| Location - Oxford | 0.16 | 0.694 | Location - Oxford | 0.08 | 0.784 | Location - Oxford | 0.00 | 0.98 |
| Location - Barking | 0.73 | 0.394 | Location - Barking | 0.15 | 0.697 | Location - Barking | 0.01 | 0.935 |
| Location - Peterborough | 4.45 | 0.037 | Location - Peterborough | 1.11 | 0.294 | Location - Peterborough | 0.83 | 0.365 |
| Location - Hertfordshire | 0.53 | 0.47 | Location - Hertfordshire | 0.31 | 0.577 | Location - Hertfordshire | 0.08 | 0.776 |
| Model N = 138, R-squared = 0.1670 | | | Model N = 220, R-squared = 0.0561 | | | Model N = 220, R-squared = 0.0420 | | |

Supplementary Table 2, Results of the regression analyses predicting child behavioural difficulties at time three from DNA methylation, child sex, their interaction, and confounders

| **Intervention Group** |  |  | | **NR3C1 DNA methylation** | | | |  | **OXYR CpG1 DNA methylation** | | | | **OXYR CpG2 DNA methylation** | | | | **FKBP5 CpG2 DNA methylation** | | | | |
| --- | --- | --- | --- | --- | --- | --- | --- | --- | --- | --- | --- | --- | --- | --- | --- | --- | --- | --- | --- | --- | --- |
|  | **Variable** | | **Beta** | | **SE** | **Std. Beta** | **p** | **Beta** | | **SE** | **Std. Beta** | **p** | **Beta** | **SE** | **Std. Beta** | **p** | **Beta** | **SE** | **Std. Beta** | **p** |  |
|  | DNA methylation | | 1.023 | | 1.901 | 0.120 | 0.593 | -0.422 | | 1.146 | 0.104 | 0.456 | -1.448 | 1.290 | -0.180 | 0.265 | -0.996 | 1.290 | -0.117 | 0.442 |  |
|  | Child sex | | 3.709 | | 1.991 | 0.233 | 0.068 | 2.625 | | 1.528 | 0.106 | 0.290 | 2.426 | 1.527 | 0.151 | 0.116 | 3.310 | 1.549 | 0.207 | 0.035 |  |
|  | DNA methylation * child sex | | -2.605 | | 2.301 | -0.235 | 0.263 | 0.237 | | 1.595 | -0.117 | 0.384 | 1.414 | 1.571 | 0.142 | 0.370 | 1.875 | 1.636 | 0.169 | 0.255 |  |
|  | PPACS T1 | | 0.234 | | 0.105 | 0.282 | 0.030 | 0.284 | | 1.295 | 0.280 | 0.005 | **0.302** | **0.083** | **0.353** | **<0.001** | **0.283** | **0.079** | **0.333** | **0.001** |  |
|  | Child age | | -0.329 | | 0.227 | -0.193 | 0.153 | -0.271 | | 0.158 | -0.117 | 0.088 | -0.258 | 0.157 | -0.160 | 0.104 | 1.875 | 1.636 | -0.174 | 0.075 |  |
|  | Number of caregivers in trial | | 2.439 | | 2.649 | 0.115 | 0.362 | 0.635 | | 1.934 | 0.034 | 0.723 | 0.628 | 1.919 | 0.030 | 0.744 | 0.283 | 0.080 | 0.017 | 0.857 |  |
|  | Time since randomisation | | 0.056 | | 0.422 | 0.016 | 0.194 | -0.185 | | 0.320 | -0.016 | 0.874 | -0.213 | 0.314 | -0.066 | 0.500 | -0.277 | 0.153 | -0.028 | 0.775 |  |
|  | Location - Camden | | -0.503 | | 2.345 | -0.030 | 0.831 | -0.253 | | 1.877 | -0.060 | 0.590 | -0.038 | 1.850 | -0.002 | 0.983 | 0.351 | 1.943 | -0.028 | 0.793 |  |
|  | Location - Hillingdon | | -6.160 | | 3.973 | -0.206 | 0.123 | -2.400 | | 3.179 | -0.105 | 0.306 | -2.227 | 3.130 | -0.069 | 0.478 | -0.090 | 0.311 | -0.147 | 0.156 |  |
|  | Location - Oxford | | 1.410 | | 3.512 | 0.054 | 0.694 | -0.244 | | 2.257 | -0.011 | 0.917 | -0.178 | 2.246 | -0.008 | 0.937 | -0.089 | 0.311 | -0.018 | 0.961 |  |
|  | Location - Barking | | -0.916 | | 6.173 | -0.020 | 0.883 | -4.247 | | 4.659 | 0.002 | 0.984 | -4.403 | 4.628 | -0.091 | 0.344 | -4.167 | 4.622 | -0.089 | 0.370 |  |
|  | Location - Peterborough | | 1.543 | | 5.973 | 0.033 | 0.797 | 3.288 | | 3.159 | 0.031 | 0.774 | 3.392 | 3.098 | 0.112 | 0.276 | 3.453 | 3.103 | 0.116 | 0.269 |  |
|  | Location - Hertfordshire | | -2.217 | | 5.790 | -0.091 | 0.470 | -6.090 | | 5.43 | -0.153 | 0.123 | -5.996 | 5.471 | -0.102 | 0.276 | -4.949 | 5.348 | -0.085 | 0.357 |  |
|  |  |  | | Model N = 65, R-squared = 0.3022 | | | | Model N = 105, R-squared = 0.205 | | | | | Model N = 105, R-squared = 0.2722 | | | | Model N = 103, R-squared = 0.3066 | | | | |
| **Control Group** |  |  | | **NR3C1 DNA methylation** | | | | **OXYR CpG1 DNA methylation** | | | | | **OXYR CpG2 DNA methylation** | | | | **FKBP5 CpG2 DNA methylation** | | | | |
|  | **Variable** | |  | |  | **Std. Beta** | **p** | **Beta** | | **SE** | **Std. Beta** | **p** | **Beta** | **SE** | **Std.**  **Beta** | **p** | **Beta** | **SE** | **Std. Beta** | **p** |  |
|  | DNA methylation | | 1.374 | | 0.214 | 0.121 | 0.524 | 2.338 | | 1.240 | 0.266 | 0.040 | 1.520 | 1.061 | 0.145 | 0.155 | 2.370 | 1.376 | 0.236 | 0.088 |  |
|  | Child sex | | -0.104 | | 2.575 | -0.004 | 0.968 | -1.339 | | 1.859 | -0.054 | 0.544 | -1.569 | 1.872 | -0.073 | 0.404 | -0.836 | 1.895 | -0.039 | 0.660 |  |
|  | DNA methylation * child sex | | -2.188 | | 2.604 | -0.159 | 0.404 | -2.608 | | 1.625 | -0.160 | 0.204 | -1.250 | 1.792 | -0.071 | 0.487 | -3.250 | 1.791 | -0.247 | 0.073 |  |
|  | PPACS T1 | | **0.543** | | **0.108** | **0.544** | **<0.001** | **0.443** | | **0.080** | **0.429** | **<0.001** | **0.442** | **0.081** | **0.470** | **<0.001** | **0.437** | **0.083** | **0.458** | **<0.001** |  |
|  | Child age | | -0.090 | | 0.230 | 0.047 | 0.699 | 0.066 | | 0.150 | 0.051 | 0.571 | 0.058 | 0.152 | 0.033 | 0.703 | 0.081 | .150 | 0.046 | 0.590 |  |
|  | Number of caregivers in trial | | -3.380 | | 3.287 | -0.117 | 0.308 | -4.755 | | 2.533 | -0.097 | 0.352 | -4.533 | 2.535 | -0.163 | 0.077 | -4.911 | 2.528 | -0.176 | 0.055 |  |
|  | Time since randomisation | | -0.293 | | 0.730 | -0.049 | 0.689 | 0.300 | | 0.485 | 0.270 | 0.702 | 0.255 | 0.486 | 0.049 | 0.602 | 0.270 | 0.487 | 0.051 | 0.580 |  |
|  | Location - Camden | | 0.533 | | 2.924 | 0.021 | 0.856 | -0.347 | | 2.229 | -0.077 | 0.429 | 0.179 | 2.221 | 0.008 | 0.936 | 0.615 | 2.206 | 0.026 | 0.781 |  |
|  | Location - Hillingdon | | 7.730 | | 5.428 | 0.171 | 0.160 | 4.802 | | 3.885 | 0.106 | 0.271 | 5.012 | 3.911 | 0.120 | 0.203 | 5.982 | 4.188 | 0.136 | 0.156 |  |
|  | Location - Oxford | | -2.327 | | 3.931 | -0.064 | 0.556 | -2.715 | | 2.699 | -0.072 | 0.433 | -2.340 | 2.719 | -0.076 | 0.391 | -1.722 | 2.733 | -0.056 | 0.539 |  |
|  | Location - Barking | | -6.841 | | 7.406 | -0.089 | 0.359 | -8.218 | | 5.617 | 0.071 | 0.424 | -7.544 | 5.639 | -0.114 | 0.184 | -7.361 | 5.620 | -0.111 | 0.193 |  |
|  | Location - Peterborough | | 7.686 | | 5.683 | 0.154 | 0.181 | 5.571 | | 3.232 | 0.250 | 0.006 | 5.689 | 3.255 | 0.152 | 0.084 | 5.790 | 3.233 | 0.154 | 0.076 |  |
|  | Location - Hertfordshire | | -0.858 | | 6.341 | -0.015 | 0.893 | -3.735 | | 4.949 | 0.003 | 0.970 | -2.766 | 4.938 | -0.048 | 0.577 | -3.620 | 4.935 | -0.962 | 0.465 |  |
|  |  |  | | Model N = 73, R-squared = 0.4198 | | | | Model N = 114, R-squared = 0.3306 | | | | | Model N = 115, R-squared = 0.3532 | | | | Model N = 114, R-squared = 0.3679 | | | | |
