# Supplementary figures and images for: Child DNA methylation in a randomised controlled trial of a video-feedback intervention to promote positive parenting and sensitive discipline (VIPP-SD)
Source: Front Child Adolesc Psychiatry. 2023 Apr 26;2:1175299. doi: 10.3389/frcha.2023.1175299 (PMC11731625; doi:10.3389/frcha.2023.1175299)

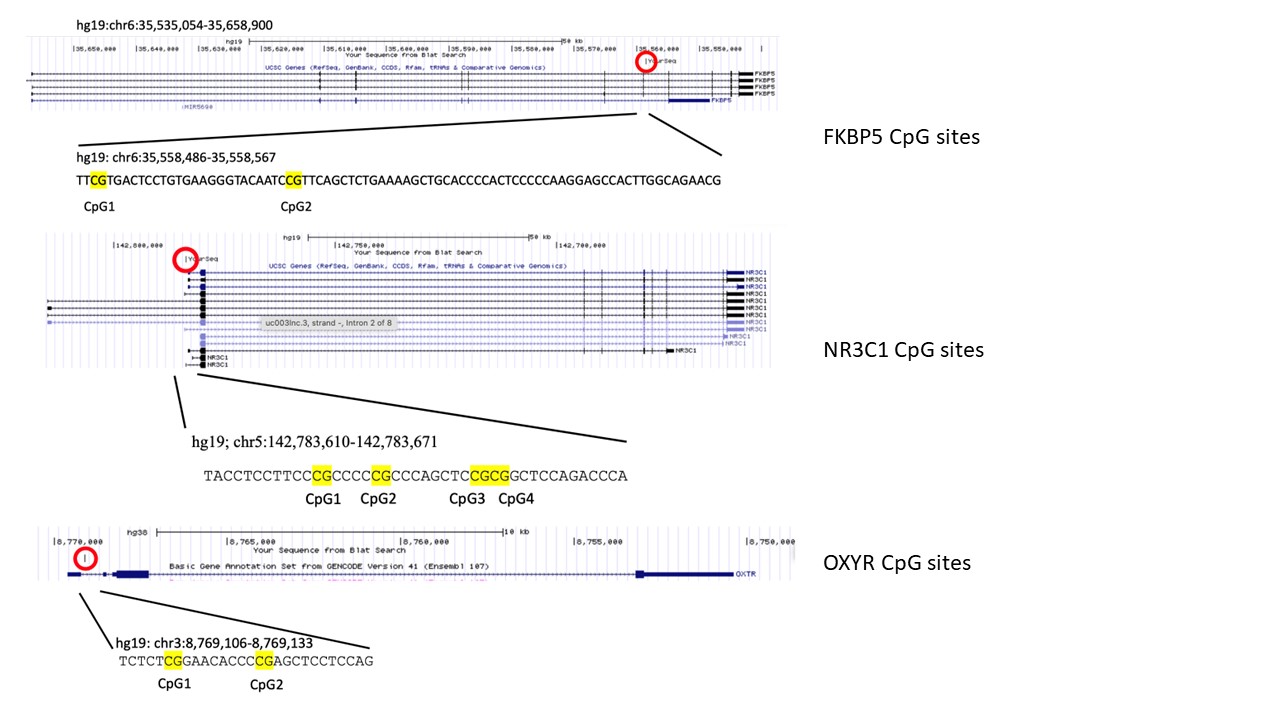

Supplement: Supplementary file 2 [file Image1.jpeg]
